# Etrog Citron (*Citrus medica*) as a Novel Source of Antimicrobial Agents: Overview of Its Bioactive Phytochemicals and Delivery Approaches

**DOI:** 10.3390/pharmaceutics17060761

**Published:** 2025-06-09

**Authors:** Arik Dahan, Ludmila Yarmolinsky, Faina Nakonechny, Olga Semenova, Boris Khalfin, Shimon Ben-Shabat

**Affiliations:** 1 Department of Clinical Pharmacology, School of Pharmacy, Faculty of Health Sciences, Ben-Gurion University of the Negev, Beer-Sheva 8410501, Israel; yludmila@post.bgu.ac.il (L.Y.); boriskh83@gmail.com (B.K.); 2 Department of Chemical Engineering, Ariel University, Ariel 4070000, Israel; fainan@ariel.ac.il (F.N.); olga.semenova@msmail.ariel.ac.il (O.S.)

**Keywords:** Etrog citron, *Citrus medica*, antibacterial properties, flavonoids, terpenes, coumarins

## Abstract

The rising prevalence of bacterial antibiotic resistance remains a significant challenge, while many existing antibacterial agents exhibit limited efficacy and notable adverse effects. Edible plants offer a promising avenue for developing novel antimicrobial drugs and preservatives. Etrog citron (*Citrus medica* L.) and its bioactive phytochemicals have demonstrated activity against various pathogenic microorganisms. However, the potential application of these compounds is hindered by factors such as poor solubility, limited bioavailability, and unclear mechanisms of action. This review consolidates key findings on the antimicrobial properties of extracts and essential oils derived from different parts of *Citrus medica*, emphasizing strategies for improving the delivery of these bioactive compounds.

## 1. Introduction

Etrog citron (*Citrus medica* L.) is an evergreen tree from the Rutaceae family cultivated in Israel, Sicily and Calabria in Italy, Corsica in France, Crete in Greece, Morocco, the United States, Southeast Asia, India, and China [1]. Forty-seven morphotypes of this plant are known according to the results of genetic analyses, and there is a large variation in their chemical contents [2]. *C. medica* is extensively used in the food, pharmaceutical, and cosmetic industries [3]. Their fruit plays an important role in Jewish rituals during the autumn harvest festival of Sukkot. The medicinal properties of the fruits, peels, pulp, juice, leaves, and flowers of *C. medica* have been described since ancient times; in fact, they are mentioned by several ancient authors, such as Pliny and Theophrastus [4]. Since the ancient and medieval use of *C. medica*, a great volume of knowledge has been accumulated regarding the numerous pharmacological properties of the plant, including its antihypertensive, diuretic, antibacterial, antifungal, anthelmintic, analgesic, antioxidant, anticancer, antidiabetic, estrogenic, antiulcer, cardioprotective, and antihyperglycemic properties [5]. The abovementioned properties of *C. medica,* including antimicrobial ones, have been considered in some reviews [3,5,6]; even so, many important aspects of their antimicrobial activities have yet to be thoroughly studied.

The extracts, essential oils, and phytochemicals of *C. medica* are of particular interest as antimicrobial agents because they are cytotoxic toward pathogenic bacteria, have low toxicity, and are relatively inexpensive and accessible. This is of particular interest due to the fact that antibiotics are not effective against life-threatening bacterial infections because of the increase in their resistance [7], while novel antimicrobial agents for combating antibiotic-resistant bacteria and food preservatives have many drawbacks [8].

Although many advantages of the antimicrobial phytochemicals of *C. medica* exist compared to antibiotics and food preservatives, many factors have substantially limited their therapeutic and industrial applications. One of them is the fact that active antimicrobial compounds cannot have the maximal effect without proper delivery to targeted locations. The objective of the present review is to extensively discuss the most prominent antimicrobial effects of the extracts and essential oils produced from various parts of *C. medica* L., as well as its phytochemicals and strategies for the delivery of these antimicrobial compounds.

## 2. Antibacterial Activity of the Extracts and Essential Oils

Multiple lines of evidence from experimental studies suggest that all parts of *C. medica* have antibacterial properties. For example, juice and ethanolic extracts of root, leaf, bark, peel, and pulp exhibit antibacterial activity against *Bacillus subtilis*, *Staphylococcus aureus*, *Enterococcus faecalis*, *Escherichia coli*, *Klebsiella pneumoniae*, *Pseudomonas aeruginosa*, and *Proteus vulgaris* [9]. The peel and pulp extracts exhibited antibacterial activity against *E. coli*, *Listeria monocytogenes*, *Pseudomonas aeruginosa*, *Staphylococcus aureus*, and *Pectobacterium carotovorum*; in addition, their inhibitory effects on the capacity of bacteria to form biofilms have been demonstrated [10].

Essential oils are multi-component mixtures of volatile compounds extracted from various parts of *C. medica* L. It has been reported that essential oil from *C. medica* L. var. sarcodactylis significantly inhibited the activity of *Listeria monocytogenes* and the formation of biofilms [11] and the activity of food-borne bacteria, such as *E. coli*, *S. aureus*, *B.*, and *Micrococcus luteus* [12]. Additionally, the essential oil derived from the peel of another variety (Greek citron) demonstrated activity against *Aspergillus niger*, *E. coli*, *Listeria monocytogenes*, *Saccharomyces cerevisiae*, *Salmonella enteritidis*, *Salmonella typhimurium*, *Staphylococcus aureus*, *Staphylococcus epidermidis*, and *Pseudomonas fragi* [13]. In addition, the essential oils from fruit peels of two *C. medica* cultivars from Southern Italy significantly inhibited *Bacillus cereus* (DSM 4313), *Bacillus cereus* (DSM 4384), *Staphylococcus aureus* (DSM 25693), *Pseudomonas aeruginosa* (ATCC 50071), and *Escherichia coli* (DSM 8579) [14]. Practically, a mixture of the essential oils, including etrog citron oil, was added to the syrup of industrial ready-to-eat fruit salads, which enhanced the microbial shelf life of the food product because of antibacterial action against *Listeria monocytogenes*, *Salmonella enteritidis*, and *Escherichia coli* [15].

The publications mentioned above do not specifically identify which phytochemicals are responsible for the antimicrobial properties. Fractions from *C. medica* L. sarcodactylis were reported to have increased antibiofilm and antibacterial activities both in vitro and in vivo after the separation and enrichment of phenol compounds [16].

## 3. Antibacterial Activity of Phytochemicals

As mentioned above, a large variation in chemical composition is present due to the diversity of chemotypes. In addition, many factors influence the chemical composition and content of extracts and essential oils (e.g., plant type or variety, geography, climate, soil features, and processing methods) [17]. In any case, antimicrobial activity cannot be referred to as a single compound because plant-active compounds often have synergic or entourage effects against both Gram-positive and Gram-negative bacteria [18].

Comparing publications devoted to the antimicrobial properties of *C. medica*, we found that flavonoids are the foremost components of this plant among the active groups. Table 1 lists the bactericidal flavonoids that were identified in the extracts of different parts of *C. medica* L. The mechanisms of their antimicrobial action are not known in many cases. Some studies have evaluated the molecular mode of action of several antimicrobial flavonoids. For example, apigenin interacts with RNA polymerase and gyrase/topoisomerase IV, nucleic acid-processing enzymes, and d-alanine ligases [19]. The mechanism of action of hesperetin against *H. pylori* is based on the inhibition of the expression of many genes related to replication, transcription, motility, and adhesion and the slowing of the expression of major virulence factors and urease [20].

Naringin promotes the formation of three kinds of reactive oxygen species, namely, hydroxy radicals, superoxide, and hydrogen peroxide, which cause apoptosis-like cell death [21]. Nobiletin and tangeretin inhibit the activities of dehydrogenase in bacterial cells, decrease the synthesis of proteins, and destroy the cell membrane [22]. The antibacterial mechanisms of quercetin are known to be better than those of other flavonoids. Quercetin may influence the permeability of bacterial cells, decrease the activity of many enzymes, disrupt cell walls, and disturb the synthesis of nucleic acids [23].

The presence of the terpenes in *C. medica* (Table 2) was mentioned in several publications [3,5] devoted to the chemical content of Etrog citron, but the antibacterial properties of these terpenes were not considered in these studies. Indeed, the antimicrobial properties of terpenes identified in *C. medica* were also found and described in many other natural sources, for example, a fungus, Xylaria Sp.YX-28 [24].

The phenolic monoterpene carvacrol was identified in the flavedo of some cultivars [25]. Carvacrol was reported to be significantly effective against 26 bacterial strains [24,26]. Carvacrol’s hydrophobic nature enables it to interact with the lipid bilayer of the bacterial membrane, leading to its expansion and destabilization [24,26]. In addition, carvacrol is able to inhibit biofilm formation by drug-resistant bacteria [27]. Other terpenes (borneol, limonene, linalool, nerol, α-pinene, β-pinene, sabinene, α-terpinene, terpinen-4-ol, and α-terpineole) of *C. medica* are constituents of essential oils that possess high antimicrobial activity [24,28].

The antibacterial mechanism of limonene against *Listeria monocytogenes* involves destruction of the cell wall and membrane, with a negative influence on the respiratory complex and ATPase [29]. A similar antibacterial mode of action was experimentally confirmed in research on *Escherichia coli* [30]. Several antibacterial mechanisms of linalool have been reported, including the disruption of cell walls in bacteria [31,32], bacterial DNA damage [33], and metabolic disorders in cells and the main metabolic pathways [34,35]. The monoterpenes α-pinene and β-pinene have toxic effects on bacterial membranes [36].

The results of the synergistic action of limonene, β-pinene, and sabinene with tetracycline were reported. They showed significant inhibition of biofilm formation of bacteria-resistant *Escherichia coli* with the following mechanisms discovered: increasing the membrane permeability of bacterial cells, disrupting the intracellular composition of microorganisms, and improving the penetration of tetracycline [37].

**Figure 1:**
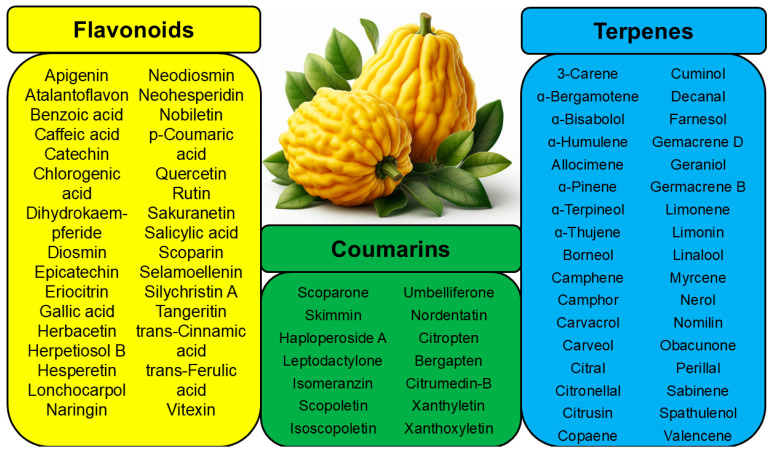
Chemical composition of *C. medica*.

Geraniol, a terpene alcohol, inhibits the activity of *Salmonella typhimurium* [38], *Escherichia coli* [39], and various oral pathogenic microorganisms [40]. The nonpolar structure of this compound allows disruption of the lipid structure of the microorganism’s cell membrane, penetrating the bacterial cell [41] and causing osmotic stress and DNA damage [42]. The chemicals identified in *C. medica* are presented in Figure 1.

Few authors studied bactericidal phytochemicals in in vivo models because it is difficult to choose an appropriate animal model according to the experimental aims and requirements. Sometimes results in vitro significantly differ from those of animal models. For example, lonchocarpol A had significant antimicrobial activity against methicillin-resistant *Staphylococcus aureus* and vancomycin-resistant *Enterococcus faecium* in vitro, but in vivo experiments were unsuccessful [43].

It was reported that a combination of colistin and apigenin was effective in killing mcr-1-positive *E. coli* in infected animals [44]. The significant bactericidal effects of hesperidin against *Aeromonas hydrophila* were demonstrated in a murine model [45]. Naringin exhibited antibacterial effects in both in vitro and in vivo experiments against *Klebsiella pneumoniae* [46]. The BALB/c mouse model of catheter-associated infection (*Staphylococcus aureus*) was used to estimate the antibacterial properties of vitexin alone and in combination with antibiotics; vitexin prevented biofilm formation [47].

**Table 1:** Antibacterial properties of the flavonoids from *C. medica*.

Compound	Structure	Part of the Plant	References
Apigenin	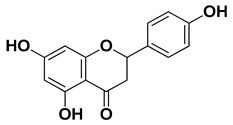	Leaves, flowers, mesocarp, endocarp	[48]
Catechin	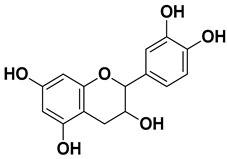	Mesocarp, endocarp, seeds, flavedo, pulp	[49]
Dihydroquercetin	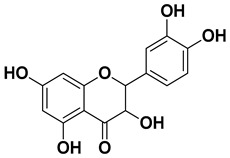	Exocarp, endocarp, seeds	[50]
Epicatechin	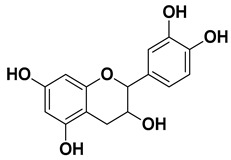	Flavedo, pulp	[51]
Herbacetin	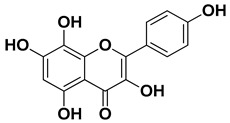	Exocarp, mesocarp, seeds	[52]
Hesperidin	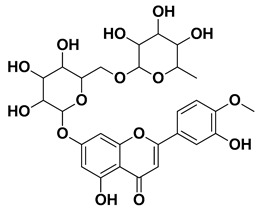	Flavedo, exocarp, endocarp, mesocarp, seeds, flowers, leaves	[53,54]
Kaempferol 3-*O*-rutinoside	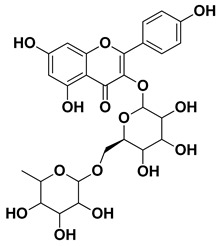	Flavedo	[55]
Lonchocarpol A	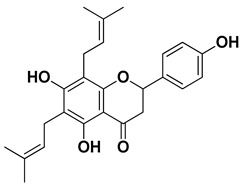	Root, stem	[56]
Naringin	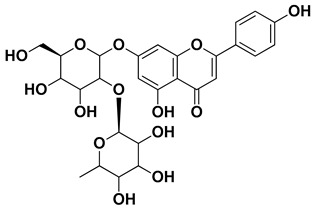	Fructus, exocarp, mesocarp, endocarp, seeds, flavedo	[57]
Nobiletin	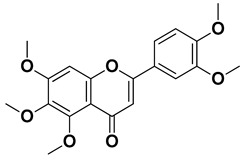	Exocarp, mesocarp, endocarp, seeds	[22]
Quercitin	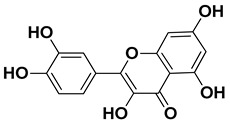	Flowers, leaves, mesocarp, endocarp	[58]
Rutin (quercetin-3-rutinoside)	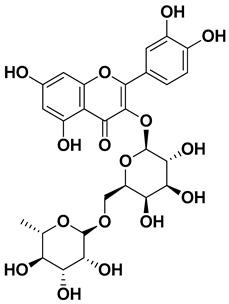	Flavedo	[59]
Tangeritin	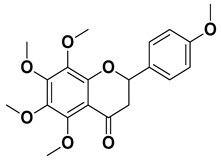	Exocarp, mesocarp, endocarp	[22]
Vitexin	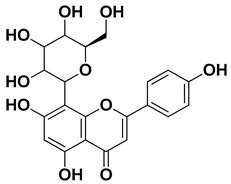	Exocarp, endocarp, seeds	[47]

**Table 2:** Antibacterial properties of the coumarins and terpenes from *C. medica*.

Compound	Structure	Part of the Plant	References
Skimmin	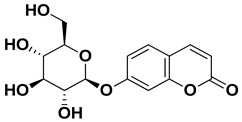	Fresh fruit	[60]
Umbelliferone (7-hydroxycoumarin)	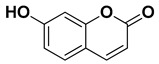	Fresh fruit	[61]
Bergapten	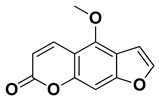	Bark, fructus	[62]
Citropten (5,7-dimethoxycoumarin)	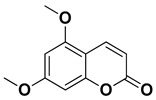	Fresh fruit, fructus	[62]
Carvacrol	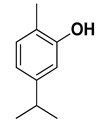	Flavedo	[24,26,27]
Borneol	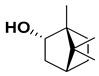	Flavedo	[24]
Limonene	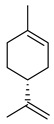	Fructus, flavedo	[24,63,64]
Linalool	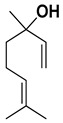	Fresh fruit, flavedo	[24,65]
Nerol	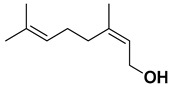	Fresh fruit, flavedo	[24,66]
α-pinene	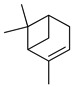	Exocarp, mesocarp, fresh fruit, flavedo, oil glands	[24,67]
β-pinene	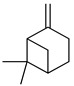	Exocarp, mesocarp, fresh fruit, flavedo, oil glands	[24,68]
Sabinene	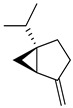	Flavedo, oil glands	[24]
α-terpinene	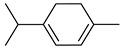	Flavedo	[24,69]
Terpinen-4-ol	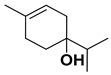	Fructus	[24,70]
α-terpineole	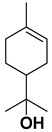	Exocarp, mesocarp, fresh fruit, flavedo, oil glands	[24,71]
Geraniol	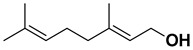	Flavedo, fructus	[38,39,40,41]

## 4. Applicability of Delivery Systems

**Figure 2:**
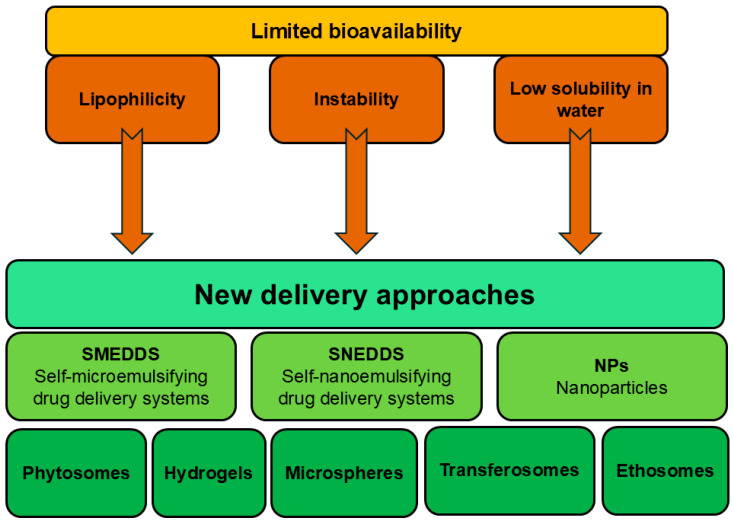
The delivery approaches that enhance the antimicrobial effects of phytochemicals, identified in *C. medica*.

The active antibacterial phytochemicals of *C. medica* are often polyphenolic compounds with low molecular weights, which have limited oral or topical bioavailability due to their lipophilicity, instability of their chemical structure, and low solubility in water [72]. The smaller the particles, the better they penetrate the cell walls of bacteria and the faster they cause the death of microorganisms [73]. In order to enhance the delivery of active compounds of *C. medica*, several new approaches are necessary, such as phytosomes, nanoparticles, self-microemulsifying drug delivery systems (SMEDDSs), and self-nanoemulsifying drug delivery systems (SNEDDSs hydrogels, microspheres, transferosomes, and ethosomes) [74]. The delivery challenges and the available approaches to deal with them are summarized in Figure 2.

Antibacterial nanoparticles are important for improving the compatibility and bioavailability of phytochemicals because of their special physical and chemical properties [75]. They take part in surface binding to bacteria and ion release with subsequent high oxidative stress; consequently, bacterial cells are not able to intensively develop gene mutations [76].

Antibacterial lipid nanoparticles consist of a lipid core surrounded by a film of surfactants that may trap active hydrophobic molecules [77]. Currently, three types of lipid nanoparticles are known: solid lipid nanoparticles, lipid nanoemulsions, and nanostructured lipid carriers [78]. Lipid antibacterial nanoparticles are biocompatible and biodegradable. Additionally, they have compact particle sizes ranging from 40 to 1000 nm, excellent payloads, and large surface areas [79].

Nanoparticles are used as encapsulants or protective shells to shield antimicrobial compounds from harsh environmental conditions [80]. Many kinds of polymers may be used in the development of drug delivery systems to create shells with the assistance of complex coacervation technology [81], freeze-drying methods [82], spray drying [83], and polyelectrolyte complexation [84].

For example, rutin did not have any significant activity against seven strains of Gram-negative or Gram-positive bacteria, but rutin-loaded mesoporous silica nanoparticles significantly inhibited them (*p* ≤ 0.05) [85]. In addition, rutin-loaded chitosan nanoparticles were more effective against *Bacillus pumilus* and *Enterococcus faecalis* than free rutin [86]. Rutin is found not only in *C. medica* but in many plants. Therefore, many kinds of nanocarriers have been developed for this flavonoid, such as nanoemulsions [87,88,89], nanoliposomes [90,91], nanocomplexes [92], and lipid nanoparticles [93]. Some methods for the preparation of nanocarriers for the delivery of rutin exist, such as encapsulation, conjugation on the surface of nanocarriers, and embedding into the structure of nanocarriers [94,95]. Nanoemulsification significantly increased the antioxidant, antibacterial, and antibiofilm activities of essential oils [96]. The most widespread metallic nanoparticles are copper oxide, aluminum oxide, iron oxide, zinc oxide, silver, and gold. The latter are the most reliable and proven [97]. For example, silver nanoparticles may influence the mitochondrial respiratory chain [98]. The cost-effective and eco-friendly way is the synthesis of metallic nanoparticles using antimicrobial compounds of *C. medica* because alkaloids, flavonoids, and terpenes have proven strong metal reduction and stabilization properties [99]. For example, it was reported that synthesis of silver nanoparticles (AgNPs) using rutin enhanced the antibacterial properties of this flavonoid against *Escherichia coli* and *Staphylococcus aureus* [100]. As per the research, the silver nanoparticles supplemented with naringenin had better bactericidal activity against *Escherichia coli*, *Vibrio cholerae*, *Staphylococcus epidermidis*, *Salmonella typhi*, *Rhodococcus rhodochrous*, *Proteus mirabilis*, and *Staphylococcus aureus* than free naringenin [101].

In another study, coatings containing ZnO nanoparticles, carvacrol, and geraniol demonstrated synergistic effect and bacteriolytic activity against *Staphylococcus aureus* and *Pseudomonas syringae* [102].

The construction of delivery systems (such as emulsions, nanostructured lipid carriers, hydrogels, and liposomes) for apigenin is an effective strategy to improve its bioavailability, but more animal and cell experiments are needed to verify these findings [103].

As mentioned, quercetin is better researched than other constituents of *C. medica.* Interestingly, zein and quercetin complexes with the encapsulation of oregano essential oil significantly improved the antibacterial properties [104].

Many carvacrol-loaded materials have been developed; several strategies have been used to produce antimicrobial composites that are more effective than carvacrol itself. For example, carvacrol-loaded chitosan nanoparticles were effective against *Staphylococcus aureus*, *Bacillus cereus*, and *Escherichia coli* [105]. The antimicrobial activity of carvacrol-loaded polyhydroxybutyrate nanoparticles against *Escherichia coli* was better than that of carvacrol [106]. Poly (lactic acid)-based fibers containing carvacrol are effective against *Listeria monocytogenes* [107]. A drug delivery system designed to combat *Staphylococcus epidermidis* biofilm formation was successfully developed by encapsulating carvacrol in poly (dl-lactide-co-glycolide) nanocapsules [108]. Compared with free carvacrol, carvacrol/clay hybrids loaded into gelling viscoelastic films were more effective against *Staphylococcus aureus* and *Escherichia coli* [108]. The bacterial strains *Pseudomonas aeruginosa*, *Staphylococcus aureus* (a methicillin-resistant strain), *Escherichia coli*, and *Enterobacter cloacae* and their pathogenic biofilms were subsequently eradicated by an oil-in-water cross-linked polymeric nanocomposite incorporating carvacrol oil [109]. Research has compared free carvacrol and liposome-encapsulated carvacrol against four strains of *Staphylococcus aureus* and *Salmonella enterica* [110]. Unfortunately, encapsulation did not increase antimicrobial activity because of the slow carvacrol release [110]. Ayres Cacciatore et al. (2020) found that while encapsulated carvacrol was less effective than free carvacrol against *Staphylococcus aureus*, *Listeria monocytogenes*, *Escherichia coli*, and several Salmonella strains, it had a milder aroma—an important advantage for the food industry [111].

A practical approach for the development of a drug delivery system by loading carvacrol against *Bacillus subtilis* was achieved as follows: cellulose nanocrystals were surface-functionalized with β-cyclodextrin, which entrapped carvacrol, succinic acid, and fumaric acid and served as bridging agents [112].

It has been demonstrated that polymeric systems (polyethylene-co-vinylacetate) containing carvacrol better inhibit the growth and biofilm formation of *Escherichia coli* and *Staphylococcus aureus* at high (37 °C) and low temperatures (4 °C), and the bactericidal effect is minimal [113]. Carvacrol may be proposed as a good antimicrobial compound for copper piping and intrauterine devices because it is electropolymerized on a copper surface [114,115]. Linalool–gold nanoparticles demonstrated better antimicrobial activities against *Staphylococcus aureus*, *Escherichia coli*, and *Leishmania tropica* than linalool alone, destroying the membranes of bacteria and causing damage to bacterial nucleic acids [116]. In addition, a mucoadhesive formulation based on methacrylate hydroxypropyl methylcellulose and methacrylate lignin encapsulated with nanostructured lipid carriers containing α-pinene was developed to monitor the release of alpha-pinene [117].

The main impediment to the use of geraniol as an antimicrobial agent is its hydrophobic nature. One of the possibilities to overcome this obstacle is developing nanoemulsions. The geraniol-based nanoemulsion E800:800 had significant antibacterial and antibiofilm activity against four *Streptococcus* spp., which are the culprits for caries development; then, it was combined with an oral rinse containing chlorhexidine, which increased the activity [118]. Another study demonstrated that nanoemulsions loaded with geraniol were effective against *Escherichia coli*, *Listeria innocua*, and *Pseudomonas lundensis* in a meat simulation medium [119].

As mentioned, animal models are rarely used. It was reported that catechin-in-cyclodextrin-in-phospholipid liposome had a significant antibacterial effect against methicillin-resistant *Staphylococcus aureus* in the Balb/c mouse model [120].

Although the phytochemicals of *C. medica* are similar to other edible plants, it has been suggested that they are likely to have minimal toxicity. However, the toxicity of materials loaded with these compounds in any form requires further investigation. Research on the safety of these products is a critical objective in the design of novel drugs and food preservatives.

## 5. Conclusions

Increasing data suggest that phytochemicals of *C. medica* could become important antibacterial agents. This review summarizes current knowledge of the antibacterial properties of the extracts, the essential oils, and the phytochemicals of *C. medica*. Taking into account that the antibiotic treatment is a rather difficult task due to biofilm formation and increasing resistance. It is important to stress that the majority of bioactive compounds present in *C. medica* are effective against drug-resistant bacteria; several phytochemicals may inhibit biofilm formation. One of the major conclusions of this review is that combating the drug-resistant bacteria could be achieved in the context of compounds of natural origin (flavonoids, coumarins, and terpenes of *C. medica*), an approach that may lead to developing new strategies in research and health policy. The flavonoid-based and terpene-based antibacterial delivery systems were discussed in this review, including strategies for improving the delivery of these compounds. However, many essential issues remain to be elucidated. In fact, the chemical compositions of many varieties are unknown, and many valuable compounds have not yet been identified. Future research on the effects of active compounds on different strains of microorganisms and their antibacterial mechanisms is needed for progress in the development of novel drug delivery systems. In addition, synergistic effects have yet to be thoroughly investigated, and the literature lacks research on animal models and clinical studies. More investigations of clinical safety and efficacy are needed to study the effectiveness of nano-formulations of *C. medica* phytochemicals.
